# Supplementary material for: Socio-economic impacts of the COVID-19 pandemic on new mothers and associations with psychosocial wellbeing: Findings from the UK COVID-19 New Mum online observational study (May 2020-June 2021)
Source: PLOS Glob Public Health. 2022 Jul 13;2(7):e0000576. doi: 10.1371/journal.pgph.0000576 (PMC10021723; doi:10.1371/journal.pgph.0000576)
Supplement: S1 Table — (PDF) [file pgph.0000576.s001.pdf]

Supporting Information

Socio-economic impacts of the COVID-19 pandemic on new mothers and associations with psychosocial wellbeing: findings from the UK COVID-19 New Mum Online Observational Study (May 2020-June 2021).

Rougeaux E, Dib S, Vázquez-Vázquez A, Fewtrell MS, Wells JCK

S1 Table. Principal Component Analysis (PCA) maternal recent psychosocial wellbeing component characteristics (N=1134)

**Variables**<br>In the last week, how much do the following statements apply to you?<br>(Response range: not at all, very little, to some extent, to a high extent)	**PCA Scoring Coefficients (Eigenvectors)**
I’ve been feeling down	0.3795
I've been feeling lonely	0.3579
I've had trouble relaxing	0.3647
I’ve become easily annoyed or irritable	0.3454
I've been feeling worried	0.3378
I’ve had trouble falling or staying asleep	0.2995
I’ve been having poor appetite	0.2162
I’ve had the opportunity to chat with my family and friends	-0.1960
I've enjoyed the weather	-0.1879
I feel able to cope with the situation	-0.2492
I’ve had time to focus on my health	-0.2330
I've had time to exercise	-0.1866

The maternal psychosocial wellbeing component identified in the PCA had an eigenvalue of 4.9 and accounted for 41% of the variance in the data. The variables and maternal psychosocial wellbeing component correlation coefficients (eigenvectors) are shown in S1 Table. These show the component has a positive association with feeling down, lonely, easily annoyed, worried, having trouble relaxing, falling asleep and having poor appetite and a negative association with having had the opportunity to chat with family and friends, enjoyed the weather, been able to cope, and had time to focus on health and to exercise. A single principal component score, our measure of maternal psychosocial wellbeing, was obtained from these coefficients.
