# Supplementary material for: Socio-economic impacts of the COVID-19 pandemic on new mothers and associations with psychosocial wellbeing: Findings from the UK COVID-19 New Mum online observational study (May 2020-June 2021)
Source: PLOS Glob Public Health. 2022 Jul 13;2(7):e0000576. doi: 10.1371/journal.pgph.0000576 (PMC10021723; doi:10.1371/journal.pgph.0000576)
Supplement: S2 Table — (PDF) [file pgph.0000576.s002.pdf]

# Supporting Information

## Socio-economic impacts of the COVID-19 pandemic on new mothers and associations with psychosocial wellbeing: findings from the UK COVID-19 New Mum Online Observational Study (May 2020-June 2021).

*Rougeaux E, Dib S, Vázquez-Vázquez A, Fewtrell MS, Wells JCK*

**S2 Table. Impact of the COVID-19 pandemic on household ability to cover expenses by maternal socio-demographic characteristics in the COVID-19 New Mum Study; % (n)**

| | | Impact on food expenses (N=3096) | | Impact on rent/mortgage payments (N=2989) | | Impact on essentials expenses (N=3085) | |
|---|---|---|---|---|---|---|---|
| | | Low | Low | Low | Moderate-major | Low | Moderate-major |
| **Maternal age (years)** | 18-25 | 27% (75) | 27% (75) | 28% (78) | 28% (77) | 25% (70) | 21% (58) |
| | 26-30 | 23% (216) | 23% (216) | 21% (189) | 23% (204) | 21% (195) | 15% (143) |
| | 31-35 | 17% (204) | 17% (204) | 17% (191) | 18% (201) | 15% (175) | 11% (131) |
| | 36+ | 18% (132) | 18% (132) | 16% (114) | 18% (130) | 14% (104) | 12% (88) |
| | *Pearson Chi2 p-value* | <0.001 | | <0.001 | | <0.001 | |
| **Household structure** | Married/civil partnership/cohabitating | 19% (567) | 19% (567) | 18% (517) | 20% (562) | 17% (489) | 13% (376) |
| | Lone parent, living on own | 29% (54) | 29% (54) | 28% (50) | 25% (44) | 26% (47) | 21% (38) |
| | *Pearson Chi2 p-value* | <0.001 | | <0.001 | | <0.001 | |
| **Household income (yearly)** | < £20,000 | 27% (62) | 27% (62) | 24% (52) | 38% (82) | 26% (58) | 35% (79) |
| | ≥£20,000 and <£30,000 | 32% (106) | 32% (106) | 24% (80) | 34% (112) | 25% (83) | 28% (93) |
| | ≥£30,000 and <£45,000 | 27% (161) | 27% (161) | 25% (143) | 24% (140) | 25% (147) | 18% (108) |
| | ≥£45,000 and <£75,000 | 19% (194) | 19% (194) | 17% (170) | 18% (179) | 17% (167) | 9% (92) |
| | ≥£75,000 and <£100,000 | 10% (43) | 10% (43) | 13% (51) | 8% (33) | 9% (36) | 4% (15) |
| | >£100,000 | 6% (22) | 6% (22) | 8% (30) | 9% (33) | 5% (19) | 2% (6) |
| | *Pearson Chi2 p-value* | <0.001 | | <0.001 | | <0.001 | |
| **Total** | | 20% (627) | 20% (627) | 19% (572) | 20% (612) | 17% (544) | 13% (420) |
