# Supplementary material for: Socio-economic impacts of the COVID-19 pandemic on new mothers and associations with psychosocial wellbeing: Findings from the UK COVID-19 New Mum online observational study (May 2020-June 2021)
Source: PLOS Glob Public Health. 2022 Jul 13;2(7):e0000576. doi: 10.1371/journal.pgph.0000576 (PMC10021723; doi:10.1371/journal.pgph.0000576)
Supplement: S3 Table — (PDF) [file pgph.0000576.s003.pdf]

Supporting Information

Socio-economic impacts of the COVID-19 pandemic on new mothers and associations with psychosocial wellbeing: findings from the UK COVID-19 New Mum Online Observational Study (May 2020-June 2021).

Rougeaux E, Dib S, Vázquez-Vázquez A, Fewtrell MS, Wells JCK

S3 Table. Impact of the COVID-19 pandemic on partner employment by household income in the COVID-19 New Mum Study; % (n)

		Changed to remote working	Put on furlough	Made redundant	Business closed/ shut down	Other impact
**Household income (yearly)**	< £20,000	3% (6)	9% (15)	6% (11)	7% (13)	12% (21)
	≥£20,000 and <£30,000	7% (21)	16% (49)	5% (15)	4% (11)	14% (44)
	≥£30,000 and <£45,000	10% (57)	15% (86)	3% (19)	4% (24)	13% (77)
	≥£45,000 and <£75,000	17% (163)	13% (128)	1% (10)	4% (37)	9% (91)
	≥£75,000 and <£100,000	19% (75)	9% (35)	1% (4)	3% (11)	9% (38)
	>£100,000	19% (70)	4% (13)	1% (4)	3% (10)	8% (27)
	Pearson Chi2 p-value					<0.001
**Total**		14% (392)	12% (326)	2% (63)	4% (106)	11% (298)
