# Supplementary material for: Socio-economic impacts of the COVID-19 pandemic on new mothers and associations with psychosocial wellbeing: Findings from the UK COVID-19 New Mum online observational study (May 2020-June 2021)
Source: PLOS Glob Public Health. 2022 Jul 13;2(7):e0000576. doi: 10.1371/journal.pgph.0000576 (PMC10021723; doi:10.1371/journal.pgph.0000576)
Supplement: S4 Table — (PDF) [file pgph.0000576.s004.pdf]

Supporting Information

Socio-economic impacts of the COVID-19 pandemic on new mothers and associations with psychosocial wellbeing: findings from the UK COVID-19 New Mum Online Observational Study (May 2020-June 2021).

Rougeaux E, Dib S, Vázquez-Vázquez A, Fewtrell MS, Wells JCK

S4 Table. Reported impact of the COVID-19 pandemic on employment and related factors in the UK COVID-19 New Mum Study

Impact reported (number of reports relating to mothers [M], partners [P], or both [B])	Examples
Inability to work from home or return to work due to lack of childcare (M=8)	*"I cannot return to work due to lack of childcare options"*<br>*"[I am] unable to work at home with an infant"*<br>*"Requires a commute to London and not possible with childcare limitations"*
Early return to work from maternity leave due to redundancy fears or partner income loss (M=7)	*"Return to work a month earlier than planned due to worry about my partners income"*
Unpaid leave to care for child(ren) (M=4)	*"I need to take unpaid leave because I can't get childcare"*
Extended maternal leave (M=2)	*"I have had to exstend my maternity for childcare as family are not available even though i wont get paid"*<br>*"Was allowed to stay at home at the end of my maternity leave to continue to care for my son who has to shield"*
Change in work hours/contract incompatible with childcare (M=4, B=1)	*"I have submitted a flexible working request but had it declined so may be forced to resign"*<br>*"Lockdown - working both full time with 2 children and no support"*<br>*"He worked very intensively for a period on ventilator design making him less available to support with childcare. He has however also been working remotely throughout which has been positive for us."*<br>*"I work in a hospital and went back mid covid, no childcare available"*
Reduced work hours or possibilities (M=16, P=99)	*"[My] Freelance work has stopped, had to extend maternity due to safety concerns"*<br>*"He's freelance and all his work has been cancelled. He's the only earner in our house so it had a huge affect"*<br>*"Supply teacher, no work"*
Salary reduction or unpaid leave (M=9, P=79)	*"My fiancee was unpaid - we were told to sheild baby as he was born poorly and they refused to pay him resulting in us really struggling and getting into debt"*<br>*"Unable to use keeping in touch working days to earn money while on maternity - avg loss of earnings in the last 3 months £3k"*<br>*"My partner has not been paid his furlough. We are borrowing money to survive"*<br>*"I work in A&E and returning to work to a completely new environment. My partner was out of work for months due to COVID and we had no money coming in as I was no longer receiving maternity payments [...]*

	[Partner] had to take a huge pay cut and now works in As a healthcare assistant in nhs he used to be a builder […] No money coming in for 4 months"<br>*"20% loss of partner wages plus my maternity pay meant we had to use some maternity savings earlier than planned. May have to take mortgage break later on in the year"*<br>*"[Partner's] Salary cut by 20% but not furloughed"*<br>*"[Partner] Did not work due to vulnerability of babies (twins) and did not get paid or furloughed"*
Facing potential redundancies (M=22, P=15)	*"Husband works agency, which are currently in talks to make a large quantity of staff redundant. Should this happen to my husband, I will be required to finish my maternity leave early and return back to work full time. As this is in a school, this causes reason for worry."*<br>*"Possible implications for our jobs after the furlough scheme has finished and the dip into recession"*<br>*"Concerned about future employment after maternity leave and partner's employment"*<br>*"Yes, can't go anywhere with my baby or see anyone. My partner is always around. I am worried about my job. I am worried about how my baby is developing"*
Changed work hours or arrangements to help with childcare (P=2)	*"Nurseries closed so we had to work from home and take care of our 20months old boy"*
Delayed job start (increased unemployment) (M=5)	*"[partner] Were due to start a new job but start date delayed indefinitely"*<br>*"[partner] Had a job offer but the start has been delayed so has been unemployed for longer"*
Difficulty finding employment (M=7, P=3)	*"[partner] Unable to fund new job for last few months as most businesses stopped interviewing"*<br>*"still unable to find gainful employment"*
Longer hours (M=2, P=33)	*"As the business owner I have had to increase my hours to keep business viable"*<br>*"[partner] Working huge number of hours a week"*<br>*"[Partner's] employer often tells him he is lucky to be in work and contacts him outside of work hours demanding he log on and do whatever is requested or be sacked. He seems to now never stop and is told he is lucky to be in a job"*<br>*"[Partner] Pay cut but working extra hours, not allowed holidays"*
Reduced debts and better finances (B=2)	*"We have managed to save money on nursery fees meaning we have paid a credit card off and are now debt free apart from car loans and mortgage"*<br>*"Had more disposable income due to being in lockdown"*
General impact on household income	*"Stress and anxiety for financial implications and the health of our families. Also we have felt very isolated and unable to seek any help for childcare"*<br>*"Difficult to not see family and friends. We are using savings to cover our monthly bills"*<br>*"kids home not enough money to feed them"*
