# Supplementary material for: Socio-economic impacts of the COVID-19 pandemic on new mothers and associations with psychosocial wellbeing: Findings from the UK COVID-19 New Mum online observational study (May 2020-June 2021)
Source: PLOS Glob Public Health. 2022 Jul 13;2(7):e0000576. doi: 10.1371/journal.pgph.0000576 (PMC10021723; doi:10.1371/journal.pgph.0000576)
Supplement: Attachment — Submitted filename: Response to Reviewers.docx [file pgph.0000576.s005.docx]

**PLOS Public Health Peer-review: Author point by point response**

**Socio-economic impacts of the COVID-19 pandemic on new mothers and associations with psychosocial wellbeing: findings from the UK COVID-19 New Mum Online Observational Study (May 2020-June 2021).**

Rougeaux E*1, Dib S1, Vázquez-Vázquez A1, Fewtrell MS1, Wells JCK1

**EDITORIAL COMMENTS**

1. Your co-authors:
Sarah Dib -sarah.dib.15@ucl.ac.uk
Mary S. Fewtrell -m.fewtrell@ucl.ac.uk
Jonathan C.K. Wells -jonathan.wells@ucl.ac.uk
,have not confirmed authorship of the manuscript. We have resent them the authorship confirmation email; however please check that the above email address for them is correct and follow up personally to ensure they confirm.

Please note that we cannot proceed your manuscript until we have received confirmations from all co-authors.

Response: all co-authors have now confirmed authorship.

2. In the online submission form you indicate that your data is not available for proprietary reasons and have provided a contact point for accessing this data. Please note that your current contact point is a co-author on this manuscript. According to our Data Policy, the contact point must not be an author on the manuscript and must be a third party. Please revise your data statement to a non-author institutional point of contact, such as a data access or ethics committee, and send this to us via return email. Please also include contact information for the third party organization, and please include the full citation of where the data can be found.

Response: statement now updated specifying that the data can be obtained through our ethics committee and their contact details (this information has also been added as an additional comment to you in the submission online).

3. Please update the completed 'Competing Interests' statement, including any COIs declared by your co-authors. If you have no competing interests to declare, please state "The authors have declared that no competing interests exist". Otherwise please declare all competing interests beginning with the statement "I have read the journal's policy and the authors of this manuscript have the following competing interests:"

Response: statement now updated in the online submission.

4. If you did not receive any funding for this study, please simply state: “The authors received no specific funding for this work.”

Response: statement now added to the submission.

Additional Editor Comments (if provided):

Title: specify period, study type

Response: information now specified in the manuscript and online submission.

Abstract: how were data collected? over what period? from text, this appears to be a convenience sample. Include N here. Give exact p-values. define "redundancy" as this term may not be familiar to international readers. [[Clarify whether the concern is for new mothers or their infants both or primarily for the long-term health and development of infants? ]]

Response: we have now specified that the data were collected online and over what period, as well as the sample number. P-values are too small to be given as exact numbers; note they should have stated p<0.001 (not 0.05) and this has now been fixed. We have changed the word redundancy for a term that would be more recognized internationally (made unemployed) here and throughout the text. We have clarified that the concern is regarding *child* long-term health and development.

Line 49: update as of early 2022

Response: now updated and reference added in (as per peer-reviewer request below), line 56.

Line 188: all these characteristics as well as other study responses are self-reported and not confirmed, correct?

Response: yes, now clarified lines 125 and 173.

Table titles: add UK to all, year

Response: now added to all tables.

Table 1: add a column with the frequency of these characteristics in the UK population overall (at line 387 you acknowledge that the sample did not reflect UK population). Please also consider possible limitations of language access as well as internet/social media access and use.

Response: column now added to table 1 (now table 2) and referred to in the Discussion section. Note that we have used census data for 2011 (which is for England & Wales) for some of the characteristics as this was the only thing available (Census 2021 data has yet to be published). Some more information on representativeness has now been added to the Results section (p.10-11) and Discussion section p.23 (including limitations relating to language and access to the online survey barriers).

Lines 392-394: here you mention that there were missing values for many variables -- these are not reflected in the tables showing the analyses; please specify the N for each analysis -- this info is in supporting info but not in the main article.

Response: sample size numbers have now been added to the results tables.

Consider reporting some of the qualitative statements from table S4 in the main article.
As noted above, this study is based on a convenience sample -- please temper conclusions accordingly.

Response: we have now added more detail to the section reporting the qualitative findings (p.17-18).

**PEER-REVIEW COMMENTS**

Reviewer #1: The authors have masterfully put together an impressive array of analyses on the socio-economic impacts of the COVID-19 pandemic on a critical group of the vulnerable population. These contributions are highly valuable. My overall concern is that the analysis process is not clear since its description is scattered with missing details of specifics of the models fit making it difficult to follow as is in the manuscript. Below are my comments and suggestions

Response: Thank you for the positive feedback and taking the time to review our work. Please find our responses to each of your comments below.

1. Line 28 indicate that it is each of the expenses listed and not either so that it is easy to understand the results as presented on line 36

Response: changes made (now line 30); we hope this is now clearer.

2. Line 30 it is not clear what is meant by socio-economic position at this point. Include a definition. I would suggest that you mention the types of models fit for each association and the rationale rather than just including the measures eg we explored the associations of i. and socio-economic position using a logistic regression model to obtain odds ratios etc

Response: thank you for the suggestion, we have now given exact variables instead of saying socio-economic position and clarified the analyses for each (now lines 32-35).

3. Line 43 whose health and development do you refer to here I would presume child? Also, the conclusion is too general. There are support packages already in place so what needs to be done is being more strategic to allocate these resources to more vulnerable populations in this case new mothers. This needs to be reflected in the conclusion provided in the abstract as it is in the last section of the paper.

Response: we have now made the conclusion more specific as suggested, however due to the 300 word limit of the abstract we were unable to make it more detailed.

4. Lines 47-49 in the introduction need to be referenced, what is the source of the information used?

Response: reference now added (reference 1) and sentence now updated to 2022 as per editor’s request.

5. I suggest that you provide Lines 120-140 as a table with a column of the indicator and the operational definition as used in the study which would be easier to follow. Include the psychosocial wellbeing on the table and then retain information on how it was defined as is in the manuscript.

Response: the information is now provided as a table (Table 1).

6. Only describe the PCA process used to construct maternal psychosocial wellbeing and move the PCA results presented in lines 148-159 after S1 table. As is, it distracts the flow of manuscript by reporting results in methods section.

Response: The PCA results now moved to after S1 Table.

7. The analyses section is a bit confusing. This section could be written better to have an easy flow of the steps taken without subdividing into sections and then repeating tests that were done across all the associations explored. Also, at this point it is not clear what models were fit for each association and the rationale behind it. It is critical to ensure that this section is described clearly for replication. Define what confounders you used. Kindly rewrite this to reflect the stepwise approach used during the analysis.

Response: the section has been rewritten (p. 8-10) and we hope it is now clearer.

8. Line 184 state the STATA license used

Response: full reference as given by STATA now added (line 214)

9. With regards to the sample characteristics section, you had stated on the methods section that disparities of socio-economic groups and stresses were assessed using proportions & chi-square test. This has not been reported here

Response: This was mistakenly put in the sample characteristics methods section and has now been removed. The Chi2 tests that were carried out are instead described in the Methods for the association analyses with expenses, employment and maternal mental health and reported in the relevant results section.

10. Review what happened to the border lines of table 4

Response: Thank you, this has now been fixed (now table 5).

11. On Line 337 kindly clarify which of your findings suggest that the long-term economic effects of the pandemic are likely to increase the impact on families further.

Response: This section has now been expanded (lines 408-410).

12. Line 389, do you mean lack of representativeness because power might allude to sample size which I don't think is the case here

Response: We meant the small size of the groups meant regression analyses could not be run, this has now been reworded for clarity.

13. Well written conclusion that links well to the findings. Align the references well, at the moment they appear disorderly

Response: Thank you for the positive feedback. The references have now been better aligned at the ends of the sentences.

14. I might be colour blind but Figure 2 the blue colours are difficult to distinguish

Response: we have now updated the figure with different colours and patterns so the groups are better differentiated and the figure is more accessible.
